# The Role of Rapid Maxillary Expansion in the Management of Obstructive Sleep Apnoea: Monitoring Respiratory Parameters—A Systematic Review and Meta-Analysis

**DOI:** 10.3390/jcm14010116

**Published:** 2024-12-28

**Authors:** Aniruddh Hariharan, Susana Muwaquet Rodriguez, Tawfiq Hijazi Alsadi

**Affiliations:** 1 Dentistry Department, Catholic University of Valencia, 46001 Valencia, Spain; anihari1602@mail.ucv.es; 2 Restorative Dentistry and Endodontics, Faculty of Medicine and Health Sciences, Catholic University of Valencia, 46001 Valencia, Spain; susana.muwaquet@ucv.es; 3 Department of Orthodontics, Faculty of Medicine and Health Science, Catholic University of Valencia, 46001 Valencia, Spain

**Keywords:** AHI, obstructive sleep apnoea, oxygen saturation, rapid maxillary expansion

## Abstract

**Background/Objectives:** Obstructive sleep apnoea (OSA) is a sleep-related breathing condition that involves the presence of episodic disruptions to the sleeping pattern due to partial or complete airway obstruction. There are a range of treatment options that exist to alleviate the symptoms of this condition including CPAP, mandibular advancement, and maxillary expansion techniques. This systematic review and meta-analysis of published articles aims to determine if rapid maxillary expansion (“RME”) is an effective treatment option in the management of OSA, using quantitative parameters of AHI and SpO_2_. **Methods:** An exhaustive review of the literature was conducted on EBSCO, PubMed, and Scopus databases. The PICO question for the systematic review was “Can rapid maxillary expansion be used as a viable treatment option using comparative AHI and SpO_2_ parameters in the management of obstructive sleep apnoea?” A meta-analysis was also performed and the software used to carry out the meta-analysis was R 4.3.2 (R Core Team (2013)). **Results:** From the initial search, 62 articles were found and a further 4 articles were obtained from manual findings. Nine articles were included in the final systematic review and meta-analysis. Eight of the studies concluded that RME was successful to varying degrees in the management of OSA across both outcome variables assessed. The meta-analysis indicated that RME is an effective treatment option in the management of adult and paediatric OSA with the improvement of both parameters. The paediatric OSA sample with specific pre-treatment anatomical presentation (clear maxillary deficiency, narrow hard palate with crossbite) showed a noted resolution of OSA following RME at a pre-pubertal age, indicating that RME can be employed as a genuine treatment option for paediatric OSA as suggested by theory. **Conclusions:** The systematic review and meta-analysis provided sufficient significant data in favour of the alternative hypothesis to indicate that RME is an effective treatment option in the management of obstructive sleep apnoea, in terms of the AHI reduction and SpO_2_ increase.

## 1. Introduction

Obstructive sleep apnoea (OSA) is a sleep-related breathing condition that can broadly be characterised as the presence of episodic disruptions to the sleeping pattern due to partial or complete airway obstruction [1]. If symptoms of excessive daytime sleepiness and subsequent impaired quality of life are observed as sequelae to OSA, the term obstructive sleep apnoea syndrome is given.

The high prevalence of this condition in modern society and variation in severity result in the necessity for a comprehensive evaluation of each individual patient in order to decide on an appropriate intervention in terms of a proposed treatment plan [2]. OSA is termed as a complex condition and is particularly important in the interests of public health because patients who suffer from OSA are also at risk of systemic complications affecting the cardiovascular, respiratory, cerebellar, and ocular level to name a few examples [3].

Though the idea is clear that the craniofacial and upper airway anatomical factors are always implicated in the pathophysiology of OSA, it is estimated that the non-anatomical risk factors are involved in about 70% of cases and can be important contributors to the severity of OSA [4]. Some of the non-anatomical pathological factors associated with OSA include a low respiratory arousal threshold and unstable ventilation control (high loop gain) [5].

A low respiratory arousal threshold (high respiratory arousal) refers to the tendency for an individual to repeatedly awaken due to a respiratory stimulus, which causes a small ventilation drive. Usually, an increased ventilation drive activates the dilation muscles in the upper airway to open it, but those with a low arousal threshold may arouse prematurely to prevent sufficient ventilation drive for dilatory muscle activation, leading to airway obstruction and OSA [6]. The subsequent poor ventilation control means that a high arousal frequency can also contribute to excessive reductions in blood gas levels (carbon dioxide and oxygen) and intermittent sleep interruptions, symptomatic of OSA. Patients with unstable ventilation control elicit an exaggerated response to reductions in blood gas levels, which leads to an increased respiratory effort that can influence the collapsibility of the airway, predisposing an apnoeic event [4].

It is important to recognise that paediatric OSA differs from adult OSA in terms of prevalence and epidemiology. For example, in paediatric OSA, one of the major implicated risk factors is adenotonsillar hypertrophy [7]. As the influence of adenoid size on OSA decreases in adolescence, this reinforces the concept that the determinants of paediatric OSA epidemiology differ from adult OSA [8]. The estimated paediatric OSA prevalence is between 1.2 and 5.7% and is believed to affect 5.7–56% of obese children [9]. Though the prevalence of obesity increases with age, it is a significant public health issue in children and represents another form of sequela leading to OSA amongst other systemic complications; so, an early, thorough screening including obesity as a recognised risk factor is essential to inform clinicians about a need for intervention. It has been theorised by some studies that the effect of body mass index (BMI) on OSA severity is more pronounced with age [10]. The increasing of BMI is associated with increasing OSA severity in children >7 years [9]. However, Uzair et al. [11] found no statistically significant differences between the BMIs of individuals in the early middle (age 35–44) and late middle age groups (age 45–60), as well as no significant correlation between BMI and OSA severity. This indicates that age may not be the predominant factor causing the fluctuation in BMI among OSA patients in the study. It is important to recognise these conflicting results regarding the relationship between BMI and OSA severity, as it reflects the uncertainty as to the exact role BMI plays in the disease progression and severity of OSA with increasing age. Additionally, it must also be noted that BMI is an inaccurate predictor of body fat content and so cannot be reliably used to assess the effect of obesity on OSA severity [11].

The plethora of treatment options available and their heterogenous nature requires the clinician to identify specific treatment options depending on the case presented. The selection process for treatment is usually influenced by the severity of each case but there is currently no agreement amongst the current literature in terms of protocol for treating by severity. This is because the main objective of OSA treatment is to relieve symptoms of sleep fragmentation, snoring, and apnoeic events during sleep, all of which can be achieved to varying extents in most available treatment options [12]. As will be discussed in further detail later, the treatment modalities for paediatric OSA differ to adult OSA cases. The most important factor to note in paediatric OSA is the potential for skeletal growth of the patient. Hence, the orthodontic correction of Class II malocclusions is possible and can be performed either through rapid palatal expansion (RPE) or by using oral appliances that allow mandibular advancement. Palatal constriction is thought to be one of the major contributing factors to OSA development due to the reduced oropharyngeal space, and so RPE can be productive by widening this space [13].

The most common treatment of choice for severe OSA is CPAP, a form of positive airway pressure (PAP). Principally, PAP therapy aims to maintain upper airway patency by delivering a continuous stream of pressurised air through a facial interface (nasal/oronasal/oral mask) [14,15]. According to the Starling resistor model, CPAP delivered with a nasal mask prevents the obstruction of the upper airway (i.e., pharyngeal collapse) as the continuous flow of air maintains a higher pharyngeal intraluminal pressure than the surrounding negative pharyngeal critical pressure [16]. However, it is important to note that the Starling resistor model assumes that only a nasal mask can be used for this theory to assume pharyngeal patency.

Maxillary transverse discrepancy is a commonly implicated risk factor in OSA onset, relating to posterior placement of the tongue that contributes to pharyngeal collapse. Maxillary expansion can facilitate a reduction in nasal resistance by increasing nasal cavity and floor volume and subsequently improving nasal airflow [17]. Maxillary expansion techniques are largely restricted to cases where maxillary deficiency is the obvious underlying cause but are a treatment option that can be used in isolation or in conjunction with other treatments (e.g., post-adenotonsillectomy in paediatric OSA) [18].

Rapid maxillary expansion (RME) is an umbrella term that includes surgically assisted rapid maxillary/palatal expansion (SARME/SARPE)*, miniscrew-assisted rapid maxillary/palatal expansion (MARME/MARPE)*, and distraction osteogenesis maxillary expansion (DOME). DOME is a variant of SARPE that is less invasive as it performs less osteotomies, which are integrated with MARPE appliances. DOME was initially performed because of the relative low success rate in achieving a midpalatal suture split in adult patients, as well as the unpredictability of skeletal expansion when osteotomies were not performed. However, DOME was recently developed as a technique in 2017 and there is limited evidence; so, for the purposes of this systematic review, this treatment intervention will not be included [14].

The fundamental concept in RME is that the widening of the midpalatal suture at the nasal floor separates the maxillary bones apart at the suture to broaden the oropharyngeal dimensions and expand the palate through the formation of new bone at the suture. The expansion consequently increases nasal cavity volume and posterior pharyngeal airway space during sleep as well as decreases nasal airflow resistance [19]. However, given that the midpalatal suture typically fuses in late adolescence, these conventional RME appliances can only be used in those OSA cases before the ossification of the midpalatal suture, where it is more responsive to expansion forces. In skeletally mature patients, conventional RME appliances are more likely to produce skeletal changes affecting the palatine bones, than directly act upon the midpalatal suture to increase the transverse dimension [18]. With MARPE and SARPE, the closed midpalatal suture can be released (split), which makes these treatment modalities ideal for mature patients whose sutures have already fused [19,20].

In SARPE, a midpalatal osteotomy is first performed in order to surgically release the closed midpalatal suture [20,21]. An expander device is then surgically placed on the midpalatal suture, and the band loops of the expander device are connected to the maxillary molars by the orthodontist. The patient is instructed to activate the expander by small amounts per day until desired growth. The expander is kept in the mouth even when expansion stops to allow the maxillary bones to fully fuse. A second osteotomy may then be performed (Le Fort I) in severe OSA cases, which produces pterygomaxillary disjunction, though its use is controversial and avoided where possible due to risk of damage to the pterygoid plexus [21]. Due to the invasive nature of treatment with multiple osteotomies, the high cost of surgery and the risk of damage to cranial nerves and the palatine artery means that SARPE is often avoided as a treatment option by many patients [14,21].

**Figure 1:**
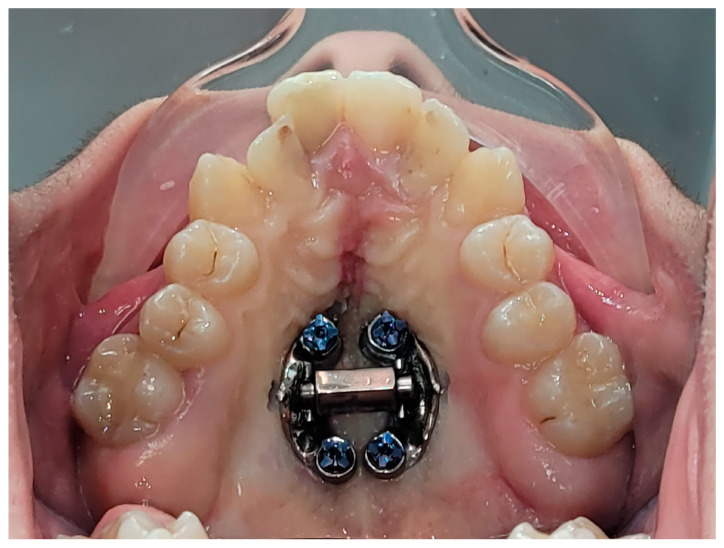
By Dr. Hijazi Alsadi. Tawfiq, Maxillary expansion appliance used in MARPE, activated by patient.

Instead, MARPE is commonly suggested as an alternative treatment, which is a non-surgical procedure that combines principles of conventional RME appliances with a custom-made skeletal anchorage device that applies mechanical forces from RME appliances directly to the basal bone via mini-implants. By directly applying the force to the maxillary bone, the mini-implants act as the anchor units whereas conventional RME appliances use teeth as anchorage for skeletal expansion. Therefore, MARPE appliances such as the one seen in Figure 1 can provide better anchorage and stability by helping to minimise unwanted dentoalveolar effects that are commonly observed with conventional RME appliances. Similarly to SARPE, the expander is activated at home by the patient by turning the MARPE screw once daily for 6 weeks, which produces incremental expansions (0.2 mm/screw turn) that release the midpalatal suture [17]. Orthodontic treatment is then initiated to close the interincisal diastema produced by the expander appliance. Similarly to SARPE, the expander is kept in the mouth for 4–6 months to allow the new bone to fully form at the suture and to prevent relapse [22].

**Figure 2:**
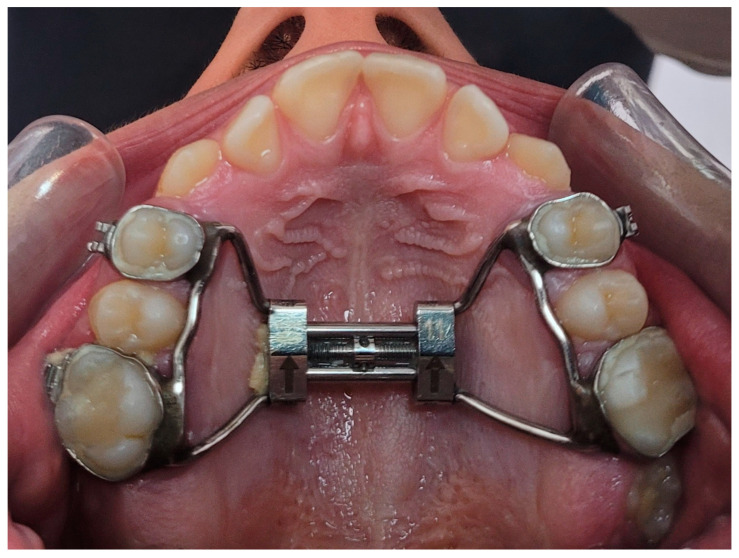
By Dr. Hijazi Alsadi. Tawfiq, Hyrax appliance.

Hyrax and Haas are types of conventional RME appliances that are to be used in cases where the midpalatal suture is patent (open); they are teeth-borne appliances that have a jackscrew in the middle of the palate that is soldered to bands that are cemented on maxillary first premolars and maxillary first molars on either side of the palate. The jackscrew is turned the first time by the orthodontist, then the patient is instructed to activate the expander by small amounts per day until desired expansion, with the frequency of activation depending on the desired amount of expansion required in the presenting case (see Figure 2). It can produce a maximum of 13 mm sutural separation and even up to 11 mm sutural separation within a short period [22,23]. Though it normally only takes a few weeks to achieve the necessary amount of expansion, patients are instructed to continue wearing the appliance for a further 6 months to allow new bone to form to produce stable expansion and to prevent expansion relapse [23,24].

Overnight polysomnography (PSG) is considered the gold standard in diagnosing OSA due to its predictive ability to provide the apnoea–hypopnea index (AHI), which is a well-established metric of OSA severity. The AHI is easily calculable from the PSG, providing a specific number of episodes of breathing obstructions per hour of sleep, which is then used to classify the severity (see Table 1). However, it is important to note that AHI is not the sole measure used by clinicians to reach a clinical diagnostic decision of OSA. It is a reliable measure due to its high reproducibility but the major disadvantage associated with its use is that it does not inform of the functional impact of the condition on the person. Other quantifiable outcome measures from the PSG that should be taken into account for a diagnosis include the oxygen desaturation index (episodes of oxygen desaturation >3% per hour of sleep) and duration of obstructive events. From the measurable outcomes from the PSG combined with prior history taking, the clinician is provided with enough information to formulate a treatment plan according to the individual case presented. The AHI will be a key metric studied in this systematic review and meta-analysis.

Currently, it is believed that maxillary expansion should be targeted at treating cases of malocclusions relating to transverse maxillary discrepancies, such as posterior crossbites where the upper teeth are placed palatally in reference to lower teeth in the transverse dimension. Alternatively, they treat cases relating to poor alignment of teeth within the upper jaw (i.e., crowding), by widening the palate to create sufficient space for proper alignment. However, the subject of improving respiratory function with reference to maxillary expansion must also be reviewed. Therefore, this systematic review and meta-analysis intends to explore to what extent maxillary expansion with RME appliances can relieve symptoms relating to OSA by expanding the palate to create space in the upper airway. Additionally, as many OSA patients fall into the older age categories due to concomitant risk factors being present, it must be noted that treatment options such as conventional RME appliances will not be effective. This is due to their mechanism of action, which is dependent on the non-fused and often pre-pubertal state of the midpalatal suture. This brings about a necessity for a treatment option that can treat the lower or upper airway obstructions causing OSA by improving the anatomical limitations by broadening the oropharyngeal dimensions. The current treatment options are focused on either extraoral appliances such as CPAP or lifestyle modifications, but recent studies have now emerged to suggest that a combined maxillofacial–orthodontist approach such as SARPE or an orthodontic approach such as MARPE could be used as viable treatment options to directly treat the cause of OSA and reduce its QoL-associated symptoms. As both treatment options are relatively novel and still emerging in the management considerations of OSA, it is acknowledged that finding clinical studies with these treatments will be difficult. Therefore, this systematic review intends to look at the available studies to determine what role RME has to play in the management of OSA.

### 1.1. Hypothesis

**Null Hypothesis (H_0_):**  *RME is not an effective treatment option in the management of obstructive sleep apnoea.*

**Alternative Hypothesis (H_1_):**  *RME is an effective treatment option in the management of obstructive sleep apnoea.*

### 1.2. Objectives

This systematic review and meta-analysis aims to determine if rapid maxillary expansion (“RME”) is an effective treatment option in the management of OSA, using quantitative parameters of AHI and SpO_2_.

## 2. Materials and Methods

**Figure 3:**
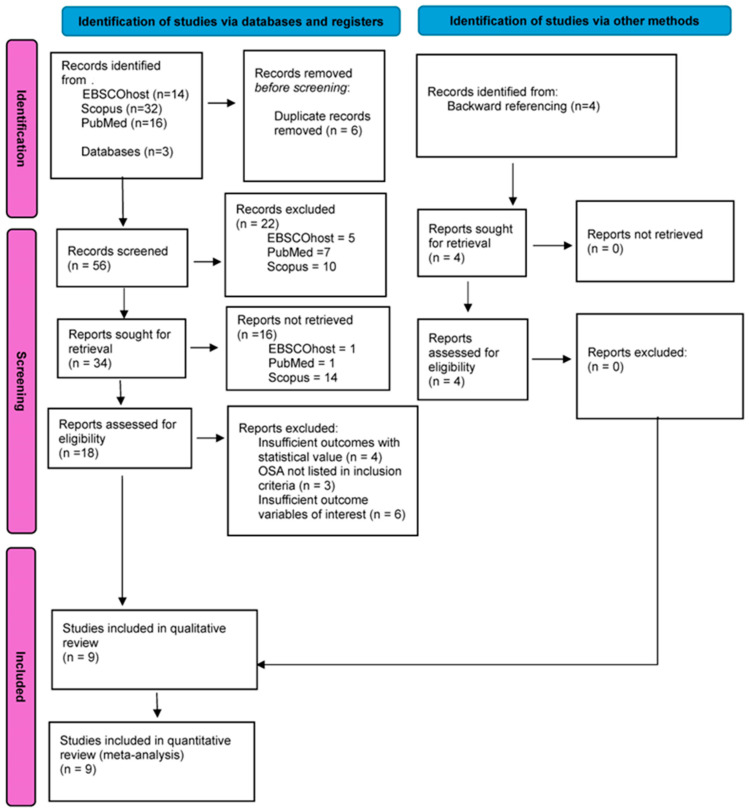
PRISMA 2020 flow diagram [26].

This systematic review was conducted in accordance with the Strengthening the Reporting of Observational Studies in Epidemiology (STROBE) Statement [25]: guidelines for reporting observational studies, and Preferred Reporting Items for Systematic reviews and Meta-Analyses Statement (PRISMA) [26]. See Figure 3

### 2.1. PICO Question Developed for the Systematic Review

In order to identify and collect studies that examined the effectiveness of maxillary expansion (RME, MARPE, or SARPE) as treatment options in the management of obstructive sleep apnoea, a PICO (population, intervention, comparison, and outcome) question was created to formulate an answerable question for the systematic review (can rapid maxillary expansion be used as a viable treatment option using comparative AHI and SpO_2_ parameters in the management of obstructive sleep apnoea?).

- Population: Patients with mixed/adolescent dentition with OSA.
- Intervention: RME.
- Comparison: AHI and SpO_2_.
- Outcome: Management of OSA.

### 2.2. Eligibility Criteria

The inclusion criteria are as follows:

- Type of patient: Patients that are treated with RME interventions. Due to the nature of this intervention as previously explained, the patients were 5–16 years old.
- Type of intervention: RME.
- Type of study: Publications in the English language; randomised controlled trials; non-randomised studies; prospective studies; clinical trials; systematic reviews; studies on humans; and no limit on study age.
- Type of outcome variables: Studies that provide data relating to AHI and oxygen saturation (SpO_2_).

Exclusion criteria:

- Type of patient: Patients that were not undergoing RME treatment.
- Type of intervention: Studies using CPAP appliance; studies using MA intervention in conjunction with RME.
- Type of study: Systematic reviews; case reports; case studies; abstracts; meta-analyses; studies on animals.
- Type of outcome variables: Scores from sleep questionnaires (e.g., ESS/BQ) or other qualitative outcome measures such as propensity for pharyngeal collapsibility. The aim is to extract only quantitative data from which we can potentially synthesise a meta-analysis.

### 2.3. Search Strategy

Subsequently, Boolean operators (AND, OR) were used alongside specific keywords chosen. The initial keywords used were “rapid palatal expansion”, “rapid maxillary expansion”, “MARPE”, “SARPE”, “MARME”, “SARME”, and “obstructive sleep apnea”. These keywords were then combined with Boolean operators, and individual searches were made across each database to obtain the most relevant studies to the PICO question, as explained below. It is important to acknowledge that MARPE and SARPE keywords were utilised with “OR” Boolean operators to widen the scope of RME-related articles gathered. The literature search was conducted from October 2023 to April 2024.

### 2.4. Keywords Used in Database Search

PubMed: ((((MARPE) OR (SARPE)) OR (MARME)) OR (SARME)) AND (sleep apnea).

EBSCOhost: Obstructive sleep apnea or obstructive sleep apnoea AND rapid palatal expansion OR rapid maxillary expansion OR MARPE OR SARPE OR MARME OR SARME.

Scopus: (sleep apnea) AND (rapid maxillary expansion) OR (MARPE) OR (SARPE).

### 2.5. Selection Process of Studies

From the advanced search, the studies that were found went through a meticulous screening procedure. Before screening any studies found, the first stage involved a pre-screening duplicate removal, meaning that any duplicates found across databases were removed (n = 6). Following this, a preliminary screening was undertaken, which was split into 2 parts. Firstly, the titles of the articles that made it to this stage were read and those that were deemed irrelevant to the topic or not part of the inclusion criteria were removed (n = 22). Then, an abstract screening was performed where the abstract, including a brief overview of the introduction, methods, results, and conclusion, was assessed and further articles were removed if deemed not relevant or failing to answer the PICO question (n = 16). The abstract screening was supervised by Dr. Hijazi Alsadi, Tawfiq to confirm eligibility of the studies, including studies considered to be most relevant with information that was considered to have obvious bias. Then, a more rigorous screening was undertaken where all remaining studies were sought for retrieval as the full texts were assessed and the relevant data were obtained. Any remaining studies at this stage that could not be retrieved for given reasons were excluded from the final systematic review (OSA not part of inclusion criteria, n = 3; insufficient outcomes of statistical value, n = 4; insufficient outcome variables of interest, n = 6). This represents the final stage of selection screening. All stages of selection screening were carried out by Aniruddh Hariharan unless otherwise specified.

### 2.6. Data Extracted

To avoid heterogeneity of the results extracted in terms of variables used and units of measurement for variables, AHI and SpO_2_ were selected as two consistent outcome variables to be analysed as a reflection of effective treatment intervention, across the studies in the meta-analysis. For the meta-analysis, the data that were extracted from the studies included mean values for outcome variables, standard deviation, and the number of participants from each study. This can be seen in Table 2. The values are given for both before the treatment and after the treatment intervention, to allow for a meta-analysis to be conducted to review and combine the results of a series of studies that analyse the effect of RME on the management of OSA.

### 2.7. Data Synthesis

The researchers developed an exhaustive review of the available literature, arriving at a final inclusion of 9 studies [22,27,28,29,30,31,32,33,34]. All of these studies presented a one-arm design of patients who were all subsidiary to RME treatment. As previously stated, the primary outcomes of the study are AHI and SpO_2_ oxygen saturation. The authors of the studies reported information on these indices before and after treatment (mean ± SD).

It should be kept in mind that the patient population in each study has a different degree of initial severity of OSA, which in principle is already a source of heterogeneity in the estimates (it is likely that patients diagnosed with the most severe OSA will experience a substantially greater reduction in AHI in terms of the absolute number of events/hour). A subgroup meta-analysis can be performed, which can differentiate patients with moderate–severe OSA from mild OSA–non-OSA patients, which was conducted for the meta-analysis estimation for AHI reduction.

A meta-analysis was performed in 2 different sections, firstly to estimate the overall measure of the effect of the reduction in AHI, and secondly to estimate the increase in SpO_2_. The model used for the meta-analysis was random effects with a Der Simonian and Laird estimator, to estimate these reductions and increases, respectively.

The I^2^ index of heterogeneity (the percentage of variability of the estimated effect that can be attributed to heterogeneity of the true effects) and the corresponding statistical test of nullity of Q were also calculated. Publication bias was explored through Funnel graphs and Egger tests, and the results of the estimates, global effect measure, and confidence intervals are plotted in the Forest graph. The level of significance that was used in the analyses was 5% (α = 0.05).

The software used to carry out the meta-analysis was R 4.3.2 (R Core Team (2013). A: A language and environment for statistical computing. R Foundation for Statistical Computing, Vienna, Austria. URL: http://www.R-project.org/).

### 2.8. Quality and Risk of Bias Assessment

The risk of bias was evaluated to analyse the methodological quality of the included 9 articles, all of which were prospective clinical studies. The Newcastle–Ottawa Scale was used as a method to evaluate the quality of the included non-randomised studies, as well as the Cochrane tool to assess the risk of bias in these non-randomised studies with interventions.

## 3. Results

**Figure 4:**
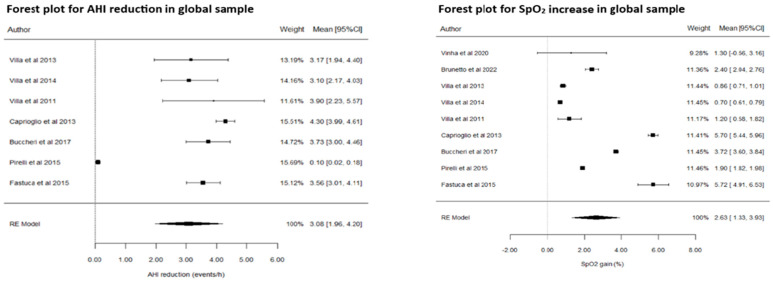
Forest Plot for AHI reduction (LEFT) and SpO_2_ increase (RIGHT) in global sample ([27,31,32,33,34]).

From the initial search, 62 articles were found and a further 4 articles were obtained from manual findings. Nine articles were included in the final systematic review and meta-analysis. Eight of the studies concluded that RME was successful to varying degrees in the management of OSA across both outcome variables assessed. The meta-analysis indicated that RME is an effective treatment option in the management of OSA with the improvement of both parameters. All data extracted from the included studies are presented in Table 2. In order to be able to thoroughly examine the effects of the interventions through the two outcome variables, the meta-analysis was divided separately into two parts (see Figure 4), firstly to estimate the overall measure of the effect of the reduction in AHI (see Table 3), and secondly to estimate the increase in SpO_2_ (see Table 4). Additionally, it is important to consider that as the heterogeneity of the meta-analysis estimates was very high in the AHI group, a subgroup analysis was proposed for this outcome variable in an attempt to reduce overall heterogeneity (see Table 5).

**Figure 5:**
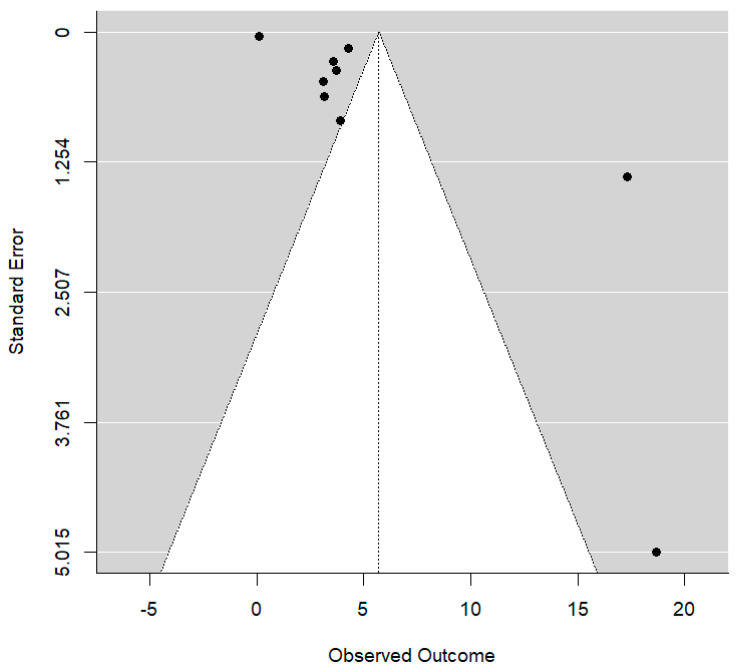
Funnel plot for bias of publications for AHI reduction in global sample.

Overall, the meta-analysis concluded that the estimated AHI reduction is 5.71 ± 1.89 events/hour. This represents a significant magnitude (*p* = 0.003) with a 95% confidence interval; i.e., the meta-analysis supports the hypothesis that RME generates a significant decrease in AHI. However, the heterogeneity of the meta-analysis estimates is exceptionally high (I^2^ > 99%). In essence, this means that the results of the articles are mixed. Villa et al. [29] and Brunetto et al. [22] provide very large changes because their population has far more severe OSA diagnoses (i.e., initial AHI). However, with the findings of the Funnel plot for bias of publications for AHI reduction in the global sample, a subgroup analysis was proposed to eliminate heterogeneity from the meta-analysis estimates. See Figure 5

The estimated AHI reduction was 17.4 ± 1.35 events/hour in the moderate–severe OSA sample. This is represented by a significant magnitude (*p* < 0.001); therefore, a conclusion can be made favouring the alternative hypothesis that MARPE and SARPE generate a significant decrease in AHI. It is also very important to note that the heterogeneity of the model has now disappeared (I^2^ = 0%), removing an obvious limitation that previously existed in the global sample.

The estimated AHI reduction in the normal–mild OSA sample is 3.08 ± 0.57 events/hour. This is represented by a significant magnitude (*p* < 0.001); therefore, a conclusion can be made favouring the alternative hypothesis that RME generates a significant decrease in AHI.

Additionally, the estimated AHI reduction in the mild OSA subgroup was 3.73 ± 0.23 events/hour. This is represented by a significant magnitude (*p* < 0.001), meaning that a conclusion can be made favouring the alternative hypothesis that RME generates a significant decrease in AHI.

With regard to the other respiratory parameter, the meta-analysis estimate of the SpO_2_ increase was 2.63 ± 0.66%. This is represented by a significant magnitude (*p* < 0.001), meaning that a conclusion could also be made favouring the alternative hypothesis that RME generates a significant increase in SpO_2_.

## 4. Discussion

Through a meta-analysis, it was our intention to integrate the information from the different studies about both respiratory parameters and thus obtain a general conclusion about the effectiveness of RME in the management of OSA. It was found that eight of the nine studies concluded that RME was successful to varying degrees in the management of OSA across both outcome variables assessed. The meta-analysis indicated that RME is an effective treatment option in the management of OSA with the improvement of both parameters. For the meta-analysis, as explained previously, due to the heterogeneity in the studies, a subgroup analysis for AHI had to be created to try and create a model that would be less prone to criticism and better to deduce accurate conclusions from. It could be concluded with confidence across the studies that RME is an effective treatment option in the management of OSA in terms of evaluating the AHI parameter. The effectiveness of RME on OSA management, however, has been shown to be case-dependent depending on initial AHI severity.

Overall, according to the meta-analysis, under all the models generated, the treatments were effective in terms of both SpO_2_ gain and AHI reduction, indicating that RME is an effective treatment option in the management of patients with OSA.

As previously discussed in the Introduction Section, AHI reduction can translate to improved airway patency and an overall reduction in OSA severity in terms of reduced symptoms for the patient, typically created by the intermittent hypoxic events that cause an exaggerated response to reductions in blood gas levels. Taking the significant magnitude associated with AHI reduction from the meta-analysis into consideration, this represents an effective outcome of treatment intervention for clinicians. Decision making for clinicians may be complicated if the magnitude of AHI reduction is not significant, as it may require a re-evaluation of the treatment plan to be considered (i.e., surgical intervention) in order to bring about a meaningful reduction.

In terms of the SpO_2_ increase, this translates qualitatively as improvements in symptoms of fatigue due to sufficient oxygen levels in sleep, where previously desaturation events were causing poor oxygen delivery to tissues and organs. Tracking SpO_2_ increases can be invaluable to the intervening clinician as other OSA-related comorbidity risk factors can be managed. For instance, the improvement of SpO_2_ coinciding with an improvement in vascular health can lead to a decision to reduce management for hypertension. An increase in SpO_2_ immediately suggests to the clinician that RME intervention has proven to be effective in maintaining a sufficient oxygen level during sleep; however, a long-term post-treatment monitoring of this parameter may prove to be important if there is a suspicion that an airway obstruction has not been properly addressed (e.g., in cases of an insignificant SpO_2_ increase post RME intervention), in which case subsequent surgical intervention may be necessary.

In terms of the suitability of RME intervention and which paediatric patients may benefit most from it, in addition to the previously discussed pre-pubertal state of the midpalatal suture, all included studies appropriately identified children with transverse maxillary deficiencies or narrow hard palates who had the potential to greatly benefit from RME intervention through widening the maxilla to correct the crossbite and occlusion.

The limitations of this systematic review and meta-analysis are largely centred around the fact that there are many sources of heterogeneity, which make it somewhat difficult to draw conclusions without there being an obvious source of variation that challenges it.

Additionally, one of the most important yet unavoidable limitations of the systematic review and meta-analysis was that we did not have any control groups to compare to the intervention being performed. This could have formed another aspect of the statistical analysis where a comparison could have been drawn for both parameters, to assess whether or not intervention is necessary according to certain patients with different status of the midpalatal suture. However, it must be acknowledged that this is unavoidable since it is ethically difficult to refrain from treating children with OSA for a period of 12 months in a study. Therefore, all the studies found were of prospective design where an intervention was always made.

Furthermore, the methods that were used for analysing the results of SpO_2_ varied in terms of their reliability. Whilst eight of the nine included studies used standard pulse oximetry to measure SpO_2_, the study of Brunetto et al. used a Bluetooth pulse oximeter typically used in home sleep testing. The main limitation with using this device is that external environmental conditions such as temperature and humidity can lead to inaccurate values recorded. Additionally, this study used an NOX T3 monitor to measure AHI instead of PSG, which is considered the gold standard in OSA laboratory diagnoses [5,12]. This presents another limitation in that this parameter is highly dependent on each participant being capable of following exact instructions given on assembling the device, as well as reproducing the same sleep conditions across both recordings. These conditions include going to bed at the exact same time, same location, and same room temperature. If any participants did not reproduce identical sleep conditions between recordings (which was not identified in the study), it likely had an impact on the AHI value recorded. The key limitation to note here is the lack of standardisation in measuring SpO_2_ across studies, which introduces variability. Brunetto et al.’s [22] study was one that contributed to the initial heterogeneity of the MA estimates, and perhaps this factor could be attributed as one of its causes.

The two major studies contributing to the heterogeneity of the overall meta-analysis are that of Vinha et al. [27] and Brunetto et al. [22]. In essence, the results are mixed and an immediate observation to explain this is that this is because their population has far more severe OSA diagnoses (i.e., initial AHI); hence, the treatment intervention produces very large changes in comparison to the remaining studies. However, it is equally important to note that with the subgroup analysis performed, the heterogeneity was able to be eliminated; hence, this did not persist as a limitation to our meta-analysis.

Additionally, it is important to note that in Vinha et al.’s study [27], no significant correlation was detected between reduced AHI and palate measurements in the overall sample; however, new significant correlations were detected when the sample was stratified according to OSA severity and there are no other obvious biases/limitations that are present in their conducted study.

In Brunetto et al.’s study [22], the main limitation that exists is that the participants of the study were not randomised; however, this was deemed to be unethical to avoid treating participants that were included in the study and were in need of treatment intervention through MARPE.

Finally, it must be acknowledged that there are a limited number of studies (n = 9) included in this systematic review and meta-analysis. This is after performing an exhaustive review of the studies available. Therefore, further clinical studies are required surrounding this treatment intervention’s effect on the studied condition as the majority of conducted studies assess the effect of this treatment intervention on malocclusions in the transversal dimensions, rather than on OSA.

## 5. Conclusions

**Null Hypothesis (H_0_):**  *There is sufficient evidence to reject the null hypothesis H_0_; RME is an effective treatment option in the management of obstructive sleep apnoea.*

AHI parameter changes:

- Sufficient significant data were provided to indicate that RME is an effective treatment option in the management of obstructive sleep apnoea in terms of AHI reduction.
- This can be seen after performing a subgroup analysis of the global OSA study sample, to combat the impact of heterogeneity on the interpretation of results.

SpO_2_ parameter changes:

- The meta-analysis estimates were significant in favour of an SpO_2_ increase, indicating that RME is an effective treatment option in the management of obstructive sleep apnoea in terms of an SpO_2_ increase.
- However, high heterogeneity indicates some uncertainty in concluding a definitive role of RME in OSA management, in terms of an SpO_2_ increase.

## Figures and Tables

**Table 1:** OSA severity classification according to AHI index and Oxygen saturation values and interpretation.

**Diagnosis by Adult OSA Severity**	**Obstructive Episodes per Hour of Sleep**
Healthy (No OSA)	<5
Mild OSA	5–15
Moderate OSA	16–30
Severe OSA	>30
**Diagnosis by Child OSA Severity**	**Obstructive Episodes per Hour of Sleep**
Healthy (No OSA)	<1
Mild OSA	1–4
Moderate OSA	5–9
Severe OSA	≥10
**Blood Oxygen Saturation (SpO_2_)%**	**Interpretation of Value**
98–100	Normal
95–97	Insufficient but tolerable (no symptoms)
90–94	Low (immediate intervention)
<90	Critical (refer to specialist)
<80	Severe hypoxia (hospitalisation)
<70	Acute danger to life

**Table 2:** Extracted data from studies for meta-analysis.

																	Intervention
	AHI								SpO_2_								
	TX			Pre-Intervention			Δ		TX			Pre-Intervention			Δ		
Author	nTX	mTX	sTX	nCT	mCT	sCT	Δm	Δs	nTX	mTX	sTX	nCT	mCT	sCT	Δm	Δs	
Vinha et al., 2020 [27]	16	14.54	19.48	16	33.23	39.54	18.69	20.06	16	94.0	1.6	16	92.7	5.4	1.3	3.8	RME (SARPE)
Brunetto et al., 2022 [22]	14	11.45	6.16	14	28.75	11.39	17.30	5.23	14	94.32	1.97	14	91.9	2.65	2.4	0.68	RME (MARPE)
Villa et al., 2013 [28]	22	2.64	3.11	22	5.81	6.05	3.17	2.94	22	97.42	1.84	22	96.6	1.47	0.86	0.37	RME
Villa et al., 2015 [29]	40	1.6	1.4	40	4.7	4.4	3.1	3	40	97.5	1.8	40	96.8	1.5	0.7	0.3	RME
Villa et al., 2011 [30]	10	2.4	2	10	6.3	4.7	3.9	2.7	10	97	2.8	10	95.8	1.8	1.2	1	RME
Caprioglio et al., 2013 [31]	14	1.4	0.6	14	5.7	1.2	4.3	0.6	14	95.5	1.6	14	89.8	1.1	5.7	0.5	RME
Buccheri et al., 2017 [32]	11	2.36	2.24	11	6.09	3.47	3.73	1.23	11	96.81	1.6	11	93.1	1.6	3.72	0	RME
Pirelli et al., 2015 [33]	23	0.3	0.9	31	0.4	1.1	0.1	0.2	23	97.2	1.5	31	95.3	1.7	1.9	0.2	RME
Fastuca et al., 2015 [34]	22	1.45	0.6	22	5.01	1.5	3.56	1.32	22	95.92	1.5	22	90.2	1.3	5.72	1.95	RME

**Table 3:** Global sample input for the AHI reduction meta-analysis.

Input for the AHI Reduction Meta-Analysis				
Author	N	GROUP	Mean	SD
Vinha et al., 2020 [27]	16	Severe	18.69	20.06
Brunetto et al., 2022 [22]	14	Moderate	17.3	5.23
Villa et al., 2013 [28]	22	Mild	3.17	2.94
Villa et al., 2015 [29]	40	Healthy	3.1	3
Villa et al., 2011 [30]	10	Mild	3.9	2.7
Caprioglio et al., 2013 [31]	14	Mild	4.3	0.6
Buccheri et al., 2017 [32]	11	Mild	3.73	1.23
Pirelli et al., 2015 [33]	23	Healthy	0.1	0.2
Fastuca et al., 2015 [34]	22	Mild	3.56	1.32

**Table 4:** Global sample input for the SpO_2_ increase meta-analysis.

Input for the SpO_2_ Increase Meta-Analysis				
Author	n	GROUP	Mean	SD
Vinha et al., 2020 [27]	16	Severe	1.3	3.8
Brunetto et al., 2022 [22]	14	Moderate	2.4	0.68
Villa et al., 2013 [28]	22	Mild	0.86	0.37
Villa et al., 2015 [29]	40	Healthy	0.7	0.3
Villa et al., 2011 [30]	10	Mild	1.2	1
Caprioglio et al., 2013 [31]	14	Mild	5.7	0.5
Buccheri et al., 2017 [32]	11	Mild	3.72	0.2
Pirelli et al., 2015 [33]	23	Healthy	1.9	0.2
Fastuca et al., 2015 [34]	22	Mild	5.72	1.95

**Table 5:** Meta-analysis results for AHI reduction in global sample, moderate–severe OSA sample, normal–mild OSA sample, and mild OSA sample and for SpO_2_ increase in global sample (from top to bottom): Weighted mean (WM), standard error (SE), 95% confidence interval, z-test (*p*-value), I^2^ index, and Cochran’s Q (*p*-value) for heterogeneity and Egger’s test (*p*-value) for publication bias.

**WM**	**SE**	**IC 95%**	**z (*p*-Value)**	**I^2^**	**Q_H_ (*p*-Value)**	**Egger (*p*-Value)**
5.71	1.89	1.99 9.43	0.003 **	99.8%	<0.001 ***	0.009 **
**WM**	**SE**	**IC 95%**	**z (*p*-Value)**	**I^2^**	**Q_H_ (*p*-Value)**	**Egger (*p*-Value)**
17.4	1.35	14.8 20.0	<0.001 ***	0.0%	0.798	---
**WM**	**SE**	**IC 95%**	**z (*p*-Value)**	**I^2^**	**Q_H_ (*p*-Value)**	**Egger (*p*-Value)**
3.08	0.57	1.96 4.20	<0.001 ***	97.7%	<0.001 ***	0.258
**WM**	**SE**	**IC 95%**	**z (*p*-Value)**	**I^2^**	**Q_H_ (*p*-value)**	**Egger (*p*-Value)**
3.73	0.23	3.29 4.18	<0.001 ***	54.7%	0.038 *	0.072
**WM**	**SE**	**IC 95%**	**z (*p*-Value)**	**I^2^**	**Q_H_ (*p*-Value)**	**Egger (*p*-Value)**
2.63	0.66	1.33 3.93	<0.001 ***	99.8%	<0.001 ***	0.964

* *p* < 0.05; ** *p* < 0.01; *** *p* < 0.001.

## References

[1] Rundo J.V. (2019). Obstructive sleep apnea basics. Clevel. Clin. J. Med..

[2] Chang H., Chen Y., Du J. (2019). Obstructive sleep apnea treatment in adults. Kaohsiung J. Med. Sci..

[3] Nakayama L.F., Tempaku P.F., Bergamo V.C., Polizelli M.U., Santos da Cruz N.F., Bittencourt L.R.A., Regatieri C.V. (2021). Obstructive sleep apnea and the retina: A review. J. Clin. Sleep Med..

[4] Antonaglia C., Passuti G. (2021). Obstructive sleep apnea syndrome in non-obese patients. Sleep Breath..

[5] Edwards B.A., Eckert D.J., Jordan A.S. (2017). Obstructive sleep apnoea pathogenesis from mild to severe: Is it all the same?. Respirology.

[6] Sands S.A., Terrill P.I., Edwards B.A., Taranto-Montemurro L., Azarbarzin A., Marques M., de Melo C.M., Loring S.H., Butler J.P., White D.P. (2017). Quantifying the Arousal Threshold Using Polysomnography in Obstructive Sleep Apnea. Sleep.

[7] Kang K.T., Chou C.H., Weng W.C., Lee P.L., Hsu W.C. (2019). Associations between Adenotonsillar Hypertrophy, Age, and Obesity in Children with Obstructive Sleep Apnea. PLoS ONE.

[8] Lumeng J.C., Chervin R.D. (2008). Epidemiology of Pediatric Obstructive Sleep Apnea. Proc. Am. Thorac. Soc..

[9] Dékány L., Molnár V., Molnár A., Bikov A., Lázár Z., Bárdos-Csenteri O., Benedek P. (2023). Analysis of possible risk factors for the severity of paediatric obstructive sleep apnoea syndrome. Eur. Arch. Oto-Rhino-Laryngol..

[10] Jasim S. (2021). Editorial for September/October Issue of AACE Clinical Case Reports. AAACE Clin. Case Rep..

[11] Uzair A., Waseem M., Bin Shahid A., Bhatti N.I., Arshad M., Ishaq A., Sajawal M., Toor Z., Ahmad O. (2024). Correlation Between Body Mass Index and Apnea-Hypopnea Index or Nadir Oxygen Saturation Levels in Patients with Obstructive Sleep Apnea. Cureus.

[12] Verbraecken J., Dieltjens M., de Beeck S.O., Vroegop A., Braem M., Vanderveken O., Randerath W. (2022). Non-CPAP therapy for obstructive sleep apnoea. Breathe.

[13] Marciuc D., Morarasu S., Morarasu B.C., Marciuc E.A., Dobrovat B.I., Pintiliciuc-Serban V., Popescu R.M., Bida F.C., Munteanu V., Haba D. (2023). Dental Appliances for the Treatment of Obstructive Sleep Apnea in Children: A Systematic Review and Meta-Analysis. Medicina.

[14] Randerath W., de Lange J., Hedner J., Ho J.P.T.F., Marklund M., Schiza S., Steier J., Verbraecken J. (2022). Current and novel treatment options for obstructive sleep apnoea. ERJ Open Res..

[15] Lance C.G. (2019). Positive airway pressure: Making an impact on sleep apnea. Clevel. Clin. J. Med..

[16] de Andrade R.G.S., Piccin V.S., Nascimento J.A., Viana F.M.L., Genta P.R., Lorenzi-Filho G. (2014). Impact of the type of mask on the effectiveness of and adherence to continuous positive airway pressure treatment for obstructive sleep apnea. J. Bras Pneumol..

[17] Kim S.Y., Park Y.C., Lee K.J., Lintermann A., Han S.S., Yu H.S., Choi Y.J. (2018). Assessment of changes in the nasal airway after nonsurgical miniscrew-assisted rapid maxillary expansion in young adults. Angle Orthod..

[18] Galeotti A., Gatto R., Caruso S., Piga S., Maldonato W., Sitzia E., Viarani V., Bompiani G., Aristei F., Marzo G. (2023). Effects of Rapid Palatal Expansion on the Upper Airway Space in Children with Obstructive Sleep Apnea (OSA): A Case-Control Study. Children.

[19] Aloufi F., Preston C.B., Zawawi K.H. (2012). Changes in the Upper and Lower Pharyngeal Airway Spaces Associated with Rapid Maxillary Expansion. ISRN Dent..

[20] Heldmaier W., Lonic D., Loeffelbein D.J. (2023). Three-Dimensional Analyses of Postoperative Effects of Surgically Assisted Rapid Palatal Expansion (SARPE) on the Soft Tissue of the Midface Region and the Upper Airway Space Using Stereophotogrammetry and Cone Beam Computed Tomography (CBCT). Am. Surg..

[21] Don Egito Vasconcelos B.C., Caubi A.F., Dias E., Lago C.A., Porto G.G. (2006). Surgically assisted rapid maxillary expasion: A preliminar study. Braz. J. Otorhinolaryngol. (Engl. Ed.).

[22] Brunetto D.P., Moschik C.E., Dominguez-Mompell R., Jaria E., Sant’Anna E.F., Moon W. (2022). Mini-implant assisted rapid palatal expansion (MARPE) effects on adult obstructive sleep apnea (OSA) and quality of life: A multi-center prospective controlled trial. Prog. Orthod..

[23] Dental E. (2022). The Clinical Benefit of Hyrax Expanders|Empire Orthodontics [Internet]. Empire Dental Specialty Group. https://empiredentalspecialty.com/how-a-hyrax-expander-works.

[24] de Oliveira Façanha A.J., Lara T.S., Garib D.G., da Silva Filho O.G. (2014). Transverse effect of Haas and Hyrax appliances on the upper dental arch in patients with unilateral complete cleft lip and palate: A comparative study. Dent. Press J. Orthod..

[25] Von Elm E., Altman D.G., Egger M., Pocock S.J., Gøtzsche P.C., Vandenbroucke J.P. (2008). The Strengthening the Reporting of Observational Studies in Epidemiology (STROBE) statement: Guidelines for reporting observational studies. J. Clin. Epidemiol..

[26] Tugwell P., Tovey D. (2021). PRISMA 2020. J. Clin. Epidemiol..

[27] Pillegi Vinha P., Rodrigues Thuler E., Veríssimo de Mello-Filho F. (2020). Effects of Surgically Assisted Rapid Maxillary Expansion on the Modification of the Pharynx and Hard Palate and on Obstructive Sleep Apnea, and Their Correlations. J. Cranio-Maxillofac. Surg..

[28] Villa M.P., Castaldo R., Miano S., Chiara Paolino M., Vitelli O., Tabarrini A., Mazzotta A.R., Cecili M., Barreto M. (2013). Adenotonsillectomy and orthodontic therapy in pediatric obstructive sleep apnea. Sleep Breath.

[29] Villa M.P., Rizzoli A., Rabasco J., Vitelli O., Pietropaoli N., Cecili M., Mar A., Malagola C. (2015). Rapid maxillary expansion outcomes in treatment of obstructive sleep apnea in children. Sleep Med..

[30] Villa M.P., Rizzoli A., Miano S., Malagola C. (2011). Efficacy of rapid maxillary expansion in children with obstructive sleep apnea syndrome: 36 months of follow-up. Sleep Breath.

[31] Caprioglio A., Meneghel M., Fastuca R., Zecca P.A., Nucera R., Nosetti L. (2014). Rapid maxillary expansion in growing patients: Correspondence between 3-dimensional airway changes and polysomnography. Int. J. Pediatr. Otorhinolaryngol..

[32] Buccheri A., Chinè F., Frat G., Manzon L. (2017). Rapid Maxillary Expansion in Obstructive Sleep Apnea in Young Patients: Cardio-Respiratory Monitoring. J. Clin. Pediatr. Dent..

[33] Pirelli P., Saponara M., Guilleminault C. (2015). Rapid maxillary expansion (RME) for pediatric obstructive sleep apnea: A 12-year follow-up. Sleep Med..

[34] Fastuca R., Meneghel M., Zecca P.A., Mangano F., Antonello M., Nucera R., Caprioglio A. (2015). Multimodal airway evaluation in growing patients after rapid maxillary expansion. Eur. J. Paediatr. Dent..

